# Selenium Biofortification Enhances Functional Bioactive Components and Nutritional Quality of Edible Mung Bean Sprouts During Germination

**DOI:** 10.3390/foods15203695

**Published:** 2026-10-17

**Authors:** Xiangxiang He, Shibin Yu, Bo Zhang, Runqiang Yang, Yizhuo Han, Hongwei Hao, Jinle Xiang

**Affiliations:** 1 College of Food and Bioengineering, Henan University of Science and Technology, Luoyang 471023, China; xianghe2009@126.com (X.H.); 18837649619@163.com (S.Y.); han0701042026@163.com (Y.H.); h138025@163.com (H.H.); 2 Henan International Joint Laboratory of Food Green Processing and Safety Control, Henan University of Science and Technology, Luoyang 471023, China; 3 School of Basic Medical Sciences, Zunyi Medical University, Zunyi 563000, China; bozhangzmu@163.com; 4 Zunyi Center for Disease Control and Prevention (Zunyi Health Supervision Bureau), Zunyi 563000, China; 5 College of Food Science & Technology, Nanjing Agricultural University, Nanjing 210095, China; yangrq@njau.edu.cn

**Keywords:** mung bean sprouts, germination period, selenium biofortification, bioactive compounds, secondary metabolites

## Abstract

This work aimed to identify the optimal edible germination period for mung bean sprouts and investigate how selenium biofortification modulates γ-aminobutyric acid (GABA), phenolic compounds, α-ketoglutarate (AKG), carotenoids, and vitamin C (VC), with a focus on improving their nutritional and functional quality as fresh edible vegetables. The results demonstrated that germination promoted the accumulation of GABA, total phenolics, total flavonoids, AKG, total carotenoids, and VC in sprouts. Nevertheless, excessively prolonged germination significantly decreased GABA, AKG, and VC contents (*p* < 0.05). Meanwhile, excessive lateral root outgrowth and full elongation of sprouts deteriorated both their functional bioactive profile and sensory edible quality. Accordingly, the 3-day germination stage was selected as the optimal harvest point for edible mung bean sprouts. Treatment with an appropriate concentration of Na_2_SeO_3_ further enhanced the contents of GABA, AKG, VC, and total carotenoids. In addition, selenium treatment significantly increased (*p* < 0.05) free total phenolic content (TPC) and total flavonoid content (TFC) while markedly reducing (*p* < 0.05) bound TPC and TFC across all tested germination stages. UPLC-ESI-MS/MS analysis revealed vitexin and isovitexin as the predominant phenolic compounds in germinated mung beans, detected in both free and bound polyphenol fractions. During germination, free vitexin and isovitexin declined, whereas bound forms of these two compounds increased. Compared with untreated controls, Na_2_SeO_3_ biofortification elevated free vitexin and isovitexin and lowered their bound fractions at different germination times. Collectively, these findings provide valuable theoretical foundations to guide the production of selenium-biofortified mung bean sprout products enriched with health-promoting bioactive ingredients for functional food applications.

## 1. Introduction

Selenium is an essential micronutrient for humans that protects against oxidative damage and regulates immune and reproductive functions [1]. The recommended daily selenium intake for adults is 50–60 μg [2]. As humans cannot synthesize selenium endogenously, low selenium contents in staple foods have led to widespread selenium insufficiency in populations across China, New Zealand, parts of Europe, and Russia. Populations with selenium deficiency exhibit increased risks of cardiovascular diseases, thyroid disorders, infectious diseases, and certain cancers [3,4,5]. Consumption of selenium-biofortified agricultural commodities represents a safe, sustainable dietary strategy to fill this nutritional gap [6]. Among available approaches, selenium biofortification via seed germination provides a simple, scalable route to produce selenium-enriched functional food ingredients [7].

Mung bean sprouts, a widely consumed fresh edible vegetable produced from germinated mung bean seeds, naturally accumulate an array of health-associated bioactive compounds during sprouting, including polyphenols, flavonoids, γ-aminobutyric acid (GABA), vitamin C (VC), carotenoids, and α-ketoglutaric acid (AKG) [8,9,10]. These metabolites confer valuable nutritional and functional properties to the edible sprouts. Phenolic compounds, VC, and carotenoids exert potent antioxidant activity and may mitigate the risk of oxidative-stress-associated chronic diseases [11,12,13]. GABA functions as an inhibitory neurotransmitter with demonstrated benefits for sleep quality and anxiety relief [14], whereas AKG presents multiple health-promoting bioactivities such as supporting muscle anabolism, accelerating wound repair, and modulating aging-related metabolism [15]. Previous investigations have confirmed that exogenous selenium application during germination successfully enriches selenium in sprouted wheat [16], foxtail millet [17], soybean [18], chickpea [1], and pea [19], while concurrently boosting the contents of GABA, polyphenols, flavonoids, VC, and carotenoids in these edible sprouts. Nevertheless, the integrated effects of selenium biofortification on selenium deposition and secondary metabolite profiles in edible mung bean sprouts remain incompletely characterized.

It is hypothesized that selenium biofortification via sodium selenite soaking could beneficially modulate selenium accumulation and the biosynthesis of functional secondary metabolites in mung bean sprouts, improving their nutritional and functional quality as edible vegetable products. To test this hypothesis, the present study quantified total selenium, organic selenium, GABA, AKG, VC, total carotenoids, total polyphenols, and total flavonoids across different germination stages of mung bean sprouts treated with Na_2_SeO_3_. UPLC-ESI-MS/MS was further applied to identify and quantify individual phenolic constituents in germinated mung beans. This work provides comprehensive understanding of how exogenous selenium biofortification shapes selenium speciation and secondary metabolite profiles within edible mung bean sprouts. Importantly, these findings provide critical theoretical support for developing selenium-enriched mung bean sprout products containing desirable functional bioactive components and facilitate the development of novel plant-based food ingredients for nutritional intervention.

## 2. Materials and Methods

### 2.1. Materials

Mung bean was bought from Henan Dazhang Industrial Co., Ltd. (Luoyang, Henan, China). 2,3-Diaminonaphthalene (DAN) and a set of polyphenol reference standards, including protocatechuic aldehyde, *p*-hydroxybenzoic acid, 2,4-dihydroxybenzoic acid, *p*-coumaric acid and rutin, was purchased from Macklin Biochemical Co., Ltd. (Shanghai, China). HPLC-grade methanol, formic acid and acetonitrile were sourced from Thermo Fisher Scientific Reagent Co., Ltd. (Waltham, MA, USA), and HPLC-grade cyclohexane was provided by Tianjin Yongda Chemical Reagent Co., Ltd. (Tianjin, China). Other chemicals used in this study were of analytical grade.

### 2.2. Mung Bean Germination Procedure

To explore the optimal germination duration for mung bean sprouts, mung bean seeds underwent soaking with distilled water at 30 °C over 10 h and were allowed to germinate for up to 7 days. Based on the screened germination time points, mung bean seeds were steeped in Na_2_SeO_3_ solutions (0, 30, 60, 90, 120, and 150 mg/L), followed by germination inside an incubator maintained at 25 °C for 3 days. Sprouted samples were sampled at intervals of one day. The raw and soaked mung beans were expressed as RMB and S1, respectively. The collected mung bean sprouts were crushed after freeze-drying and preserved for future use.

### 2.3. Extraction and Determination of Selenium

Inorganic selenium was extracted according to [20] with slight modifications. Briefly, sprouted mung bean powder was dispersed in ultrapure water, sonicated and then centrifuged for 15 min (10,000 rpm) to obtain the inorganic selenium extract. An aliquot of 5 mL from the resulting extract was combined with 10 mL HNO_3_-HClO_4_ mixed solution (*v*/*v*, 9:1) for digestion. Following overnight digestion, 6 M HCl was introduced into the digested mixture, and the system was heated until the volume was reduced to approximately 2 mL, yielding the inorganic selenium solution ready for subsequent determination. For the test of total selenium, sprouted mung bean powder was digested by adding mixed acids (HNO_3_ + HClO_4_), and the digestion steps were the same as for inorganic selenium. Determination of selenium content was carried out by fluorescence spectrophotometry according to Cheng et al. [21]. The resulting selenium solution was mixed with 2,3-Diaminonaphthalene (DAN) reagent, followed by cyclohexane extraction prior to fluorescence intensity measurement. A calibration curve was constructed using Na_2_SeO_3_, and analytical outcomes were reported in mg/kg. Organic selenium content was computed as the difference between total selenium content and inorganic selenium content.

### 2.4. Extraction and Quantitative Analysis of γ-Aminobutyric Acid (GABA)

GABA extraction was carried out following an established protocol reported elsewhere [22]. Briefly, defatting of sprouted mung beans was performed twice with n-hexane. Afterwards, the material was extracted two times with 20 mL 80% methanol for 1 h per extraction. Following centrifugation of the extracts, the supernatant was collected and reserved for the subsequent measurement of GABA. Quantitative analysis of GABA was achieved on a triple-quadrupole MS system fitted with a C18 column (100 mm × 3 mm). Mobile phase A consisted of formic acid aqueous solution, and acetonitrile was assigned as mobile phase B, operating at a flow rate of 0.2 mL/min. Full details regarding gradient program and MS parameters were referenced from our previous work [23]. Column temperature was kept at 30 °C, and 10 μL sample aliquots were injected. MRM acquisition in ESI^+^ positive mode was applied for GABA quantification, and the data were presented in mg/kg.

### 2.5. Extraction of Polyphenols

Extraction of free and bound phenolics was carried out based on our prior reported approach [24]. Free phenolics were isolated using the same workflow adopted for GABA measurement. To obtain bound phenolics, the solid residue leftover from free-phenolic extraction was mixed with 2 M NaOH. The mixture was shaken for 2 h, and then acidified with 6 M HCl to reach pH 1.5–2.0. Post-centrifugation supernatant was extracted three times with ethyl acetate. Collected organic phases were evaporated to dryness employing a rotary evaporator (RV 8 V-C, IKA-Werke GmbH & Co. KG, Staufen, Germany). The residues were redissolved in 50% methanol for the preparation of bound phenolic extracts.

### 2.6. Quantification of Total Phenolic and Total Flavonoid Contents

The Folin–Ciocalteu assay was adopted for the measurement of total phenolic content (TPC) [25]. Briefly, sample extract solutions were combined with Folin–Ciocalteu reagent (1:2) for a 5 min reaction period. Na_2_CO_3_ solution was then introduced, and the mixed system underwent incubation (25 °C) for 90 min. Spectrophotometric absorbance was measured at 750 nm, and TPC was presented in ferulic acid equivalents (mg FAE/100 g DW). Total flavonoid content (TFC) was determined following the aluminum-chloride colorimetric method [24]. The sample extracts reacted with NaNO_2_ and AlCl_3_, followed by absorbance measurement at 510 nm. Isovitexin was applied as reference standard.

### 2.7. Identification and Quantification of Phenolic Compounds

Identification and quantitative measurement of phenolic constituents were performed following approaches described in our previous study [17]. The UPLC-MS/MS apparatus and separation column used here were consistent with the configuration for GABA analysis. Both gradient elution conditions and mass spectrometer settings were sourced from our formerly reported method [26]. Phenolic candidates were recognized either through retention-time alignment with standard substances or by cross-referencing UV and MS-MS spectral features against existing literature data. Where available, corresponding standards were utilized for quantification. For phenolics without accessible standards, analog or aglycone equivalents were employed for quantitative calculation.

### 2.8. Extraction and Quantification of α-Ketoglutaric Acid

The extraction of α-ketoglutaric acid (AKG) was conducted with slight modifications based on a previously established protocol [27]. Briefly, sprouted mung bean samples were homogenized with distilled water and incubated at 60 °C for 2 h. After centrifugation, the resulting supernatant was harvested for subsequent AKG detection. The UPLC-MS/MS instrument and chromatographic column adopted in this assay were consistent with those used for GABA quantification. The mobile phase system consisted of 0.1% formic acid aqueous solution (phase A) and 0.1% formic acid acetonitrile solution (phase B). The gradient elution program was configured as follows: 95% phase A from 0 to 3.5 min, a linear decrease from 95% to 10% phase A during 3.5–5 min, constant 10% phase A from 5 to 6.5 min, and a linear rise back to 95% phase A within 6.5–7.5 min. The column temperature, flow rate and sample injection volume were maintained at 28 °C, 0.2 mL/min and 10 μL, respectively. Negative multiple reaction monitoring (MRM) mode was adopted for mass spectrometric measurement. Major parameters included capillary voltage (1.6 kV), cone voltage (20 V), ion source temperature (150 °C), desolvation gas temperature (500 °C), desolvation nitrogen flow (800 L/h), and cone helium gas flow (20 L/h). The characteristic quantitative ion transition of AKG was determined at *m*/*z* 145/101. An external standard calibration method was applied for AKG quantitative analysis, and the final content results were normalized and presented in mg/kg.

### 2.9. Determination of Total Carotenoid Content

The extraction of carotenoids followed our previously reported method [24]. The sample was mixed with acetone/ethanol solution (1:1, *v*/*v*), extracted in a water bath shaker for 2 h, and the temperature was maintained at 40 °C. The supernatant after centrifugation was the test solution. Spectrophotometric measurement at 450 nm was employed to analyze total carotenoid content (TCC), and the obtained data were expressed as milligrams per kilogram (mg/kg).

### 2.10. Determination of Vitamin C (VC) Content

The content of VC was quantified by HPLC in accordance with the Chinese National Standards (GB5009.86-2016) [28]. In brief, the sprouted mung bean sample was homogenized with metaphosphoric acid solution and subjected to ultrasonic extraction for 5 min. The mixture was centrifuged at 4000 rpm for 5 min, and the resulting supernatant was collected as the final test solution. VC quantification was conducted using an HPLC system fitted with a C18 chromatographic column (250 × 4.6 mm). The column temperature, flow rate, detection wavelength, and injection volume were set to 25 °C, 0.7 mL/min, 245 nm, and 20 μL, respectively. The mobile phase A was a mixed solution of potassium dihydrogen phosphate and cetyltrimethylammonium bromide, and the mobile phase B was methanol, which was eluted according to the ratio of A:B of 98:2. Results were quantified as mg/kg.

### 2.11. Statistical Analysis

All experiments were performed in triplicate biological replicates, and all quantitative results were expressed as mean ± standard deviation (SD). Statistical computations were performed using IBM SPSS Statistics 26.0 (IBM Corporation, Armonk, NY, USA). Two-way ANOVA was applied to evaluate the interactive and individual effects of two independent variables (germination time and sodium selenite soaking concentration). For datasets involving only a single experimental factor, one-way ANOVA was adopted. Duncan’s multiple-range test was used for post hoc mean comparisons. Differences were considered statistically significant at *p* < 0.05. Different lowercase letters indicate significant differences among means within the same comparison set, while asterisks (*) denote significant differences between selenium-treated groups and the corresponding control. All figures were generated with OriginPro 2022 (OriginLab Corporation, Northampton, MA, USA).

## 3. Results

### 3.1. Effect of Germination Time on the Content of Secondary Metabolites in Mung Bean Sprouts

#### 3.1.1. Effect of Germination Time on GABA Content in Mung Bean Sprouts

**Figure 1:**
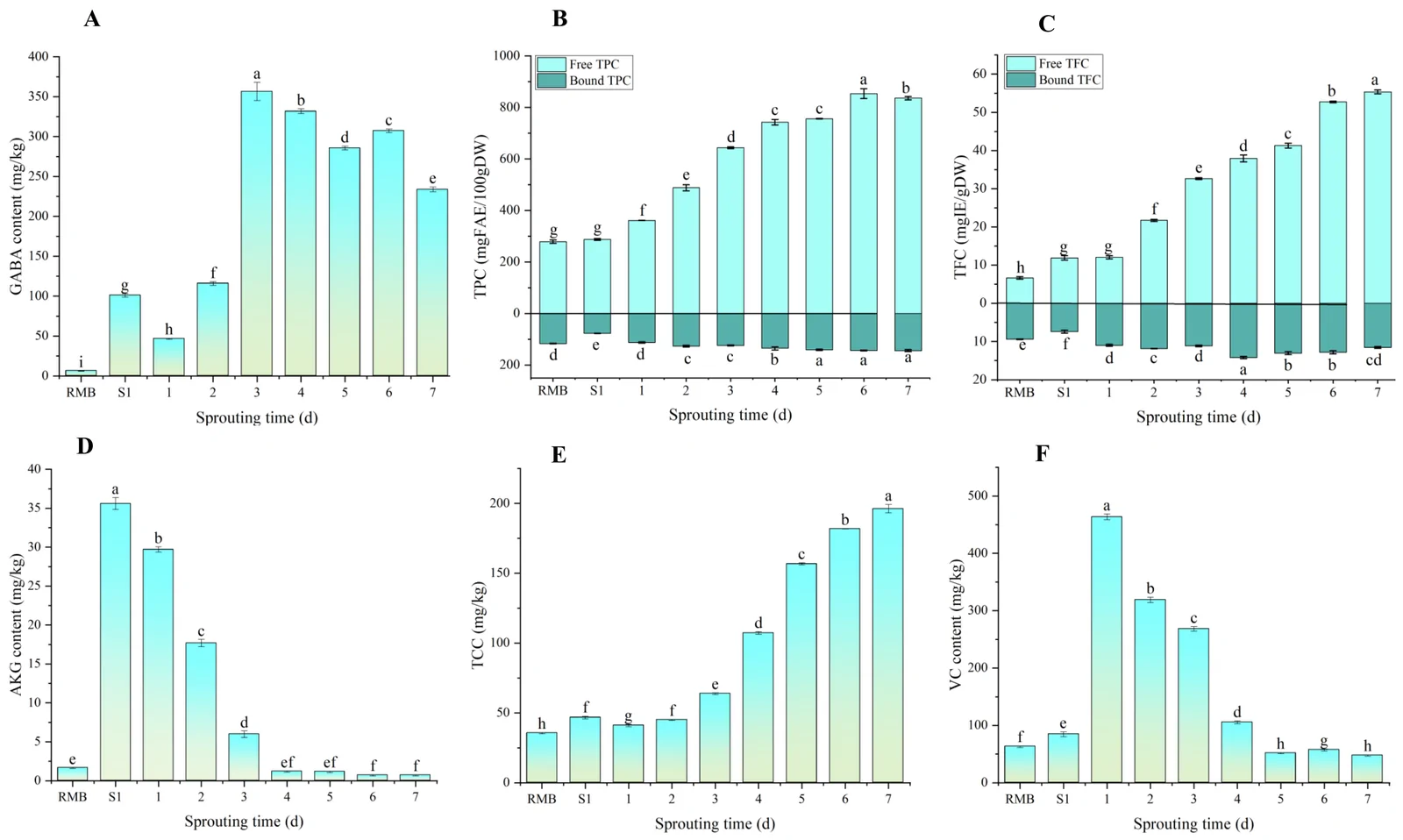
Effects of germination time on GABA content (**A**), TPC (**B**), TFC (**C**), AKG content (**D**), TCC (**E**), and VC content (**F**) of sprouted mung beans. In panels (**B**,**C**), light cyan bars represent free fractions, and dark cyan bars represent bound fractions. Different lowercases in the same histogram indicate statistically significant differences (*p* < 0.05). RMB, raw mung bean; S1, soaked mung bean.

Variations in GABA content within mung bean sprouts under different germination periods are presented in Figure 1A. GABA concentrations exhibited an initial increase followed by a decrease as germination time was extended. Compared with raw mung beans (RMBs), sprouted mung beans achieved statistically significant elevation in GABA content (*p* < 0.05). This finding can likely be attributed to the activation of glutamate decarboxylase (GAD) during germination; this enzyme catalyzes the decarboxylation of glutamate to produce GABA. In addition, ongoing protein degradation releases glutamate, which serves as sufficient precursor material for GABA synthesis [29]. Significant increases in GABA concentrations after germination have also been reported in paddy rice, brown rice, quinoa, and coix seed [30,31,32]. The GABA content of soaked mung beans (S1) was significantly (*p* < 0.05) higher than that of mung beans germinated for 1 d. This phenomenon may arise from hypoxia-induced cell acidification during soaking, which effectively increases glutamate decarboxylase (GAD) activity [33].

#### 3.1.2. Effect of Germination Time on TPC of Mung Bean Sprouts

As germination proceeded, free TPC showed an initial increase followed by a gradual decline. In contrast, bound TPC first rose and then remained statistically stable with no significant differences (*p* > 0.05), as visualized in Figure 1B. The above changing trends were consistent with a previous study [34], which reported that germination effectively enhances both free and bound phenolic contents in spelt grains. Such metabolic variation is plausibly caused by the germination-induced activation of phenylalanine ammonia-lyase (PAL), a rate-limiting enzyme for phenolic biosynthesis. The increased PAL activity further promotes the biosynthesis and accumulation of phenolic metabolites in sprouted seeds. However, the bound phenolic content of mung beans was markedly lowered (*p* < 0.05) after the soaking process. Similar results were reported by Cao et al. [35], that the bound TPC in wheat decreased significantly after soaking, owing to cellulase- and xylanase-mediated cell wall degradation that releases bound polyphenols.

#### 3.1.3. Effect of Germination Time on TFC of Mung Bean Sprouts

Over the tested germination time points, prolonged germination induced steady accumulation of free TFC. For bound TFC, its level increased at the early stage and subsequently declined (Figure 1C). Such variation patterns were comparable to the findings from Li et al. [36], where pronounced increases in free and bound flavonoid contents were detected in germinated sorghum. The rise in flavonoid contents may be linked to the activation of the phenylpropanoid pathway and its associated enzymes, chalcone isomerase (CHI) and PAL, during germination [37]. However, the bound TFC of mung beans decreased by 21.28% after soaking, which may be attributed to the release of bound TFC during the soaking process.

#### 3.1.4. Effect of Germination Time on AKG Content of Mung Bean Sprouts

After soaking, AKG content in mung beans peaked at 21.76-fold that of RAW, and prolonged germination markedly reduced AKG concentrations (*p* < 0.05), as shown in Figure 1D. AKG could be produced by the oxidative deamination of glutamate catalyzed by glutamate dehydrogenase, while AKG could be decarboxylated by AKG dehydrogenase to form succinyl CoA and CO_2_ in the tricarboxylic acid (TCA) cycle [38]. The significant (*p* < 0.05) increase in AKG after soaking may be due to the insufficient energy supply caused by the hypoxic environment, which inhibited the process of metabolizing AKG into succinyl-CoA and CO_2_. At the same time, soaking combined with hypoxic stress might elevate the activity of glutamate dehydrogenase, thereby promoting the accumulation of AKG. Such observations agreed with Lee et al. [27], where significant elevation of AKG content was detected in rice subjected to hypoxic stress. During subsequent germination, cellular energy supply recovered, and TCA cycle flux resumed, leading to AKG depletion.

#### 3.1.5. Effect of Germination Time on TCC of Mung Bean Sprouts

The effect of germination time on TCC is shown in Figure 1E. With the increase in germination time, TCC decreased first and then increased continuously, and the TCC of mung bean sprouts was significantly higher than RMBs. A similar trend was reported by Zhang et al. [39], that the carotenoid concentrations in rapeseed decreased at the early germination stage and then rose markedly. The decrease in TCC may be due to the production of a large number of free radicals and lipid oxidation products in the early stage of germination, which could quickly consume the accumulated carotenoids [40]. As documented in a previous study [13], phytoene synthase (PSY) represents the primary rate-limiting enzyme for carotenoid synthesis. The rise in total carotenoid content (TCC) may result from increased PSY activity during germination.

#### 3.1.6. Effect of Germination Time on VC Content of Mung Bean Sprouts

The effect of germination time on VC content is shown in Figure 1F. As germination proceeded, VC content first increased and then decreased, peaking at 463.73 mg/kg on day 1. Similar results have been reported in germinated chickpea [41], germinated chia [12], and germinated soybean [42]. In plants, the biosynthesis of VC is mainly carried out via the L-galactose pathway [43]. The VC accumulation in mung bean sprouts may relate to enhanced L-galactono-1,4-lactone dehydrogenase activity; this key enzyme in the L-galactose pathway catalyzes the oxidation of L-galactono-1,4-lactone to VC [44].

#### 3.1.7. Effect of Germination Time on the Morphology of Mung Bean Sprouts

**Figure 2:**
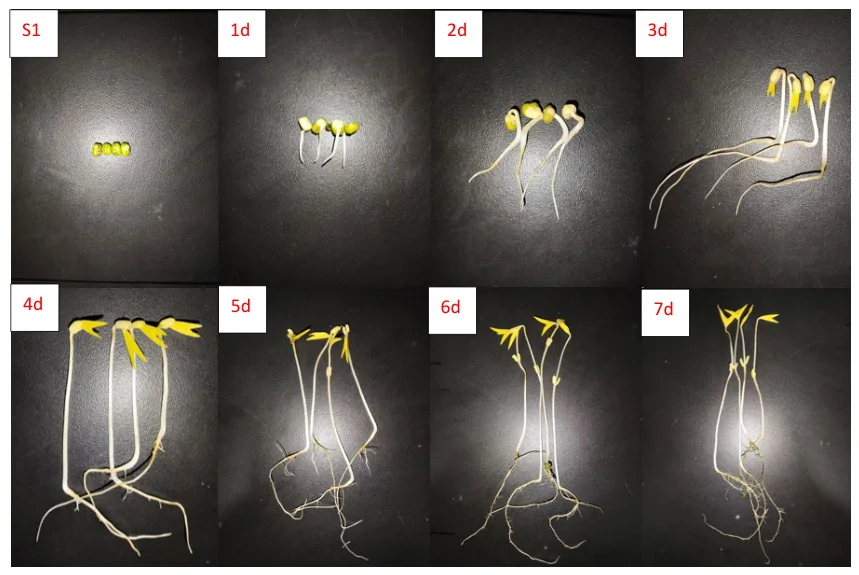
Effect of germination time on the morphology of sprouted mung beans. S1, soaked mung bean. 1d, 2d, 3d, 4d, 5d, 6d, and 7d represent mung beans germinated for 1, 2, 3, 4, 5, 6, and 7 days, respectively.

The morphology of mung bean sprouts under different germination times is shown in Figure 2. With the increase in germination time, the hypocotyls and radicles of mung bean sprouts gradually elongated, and the germs emerged. When the germination time reached the fourth day, the lateral roots of the mung bean sprouts began to grow in large numbers, and the germs were completely exposed. Cotyledons and hypocotyls are considered to be edible parts of these bean sprouts [45]. Excessive growth of lateral roots and plumules impairs the sensory quality of mung bean sprouts, and extended germination causes losses of GABA, AKG, and VC. Therefore, the germination cycle of mung bean sprouts was set to 3 d.

### 3.2. Effect of Na_2_SeO_3_ Soaking Concentration on the Content of Secondary Metabolites in Mung Bean Sprouts

#### 3.2.1. Impacts of Na_2_SeO_3_ Soaking Concentrations on Total-Selenium and Organic-Selenium Accumulations in Mung Bean Sprouts

**Figure 3:**
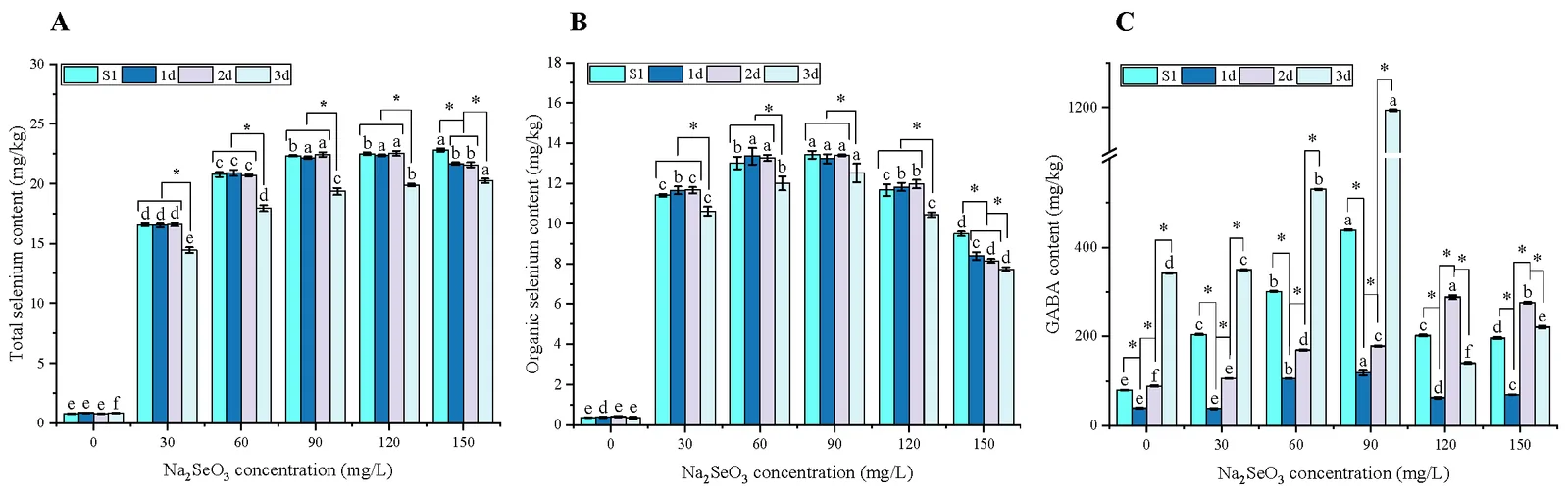
Effect of Na_2_SeO_3_ concentration on total selenium content (**A**), organic selenium content (**B**), and GABA content (**C**) of sprouted mung beans. Within the same Na_2_SeO_3_ concentration, different lowercase letters indicate significant differences (*p* < 0.05) among different germination stages. Asterisks (*) denote significant differences (*p* < 0.05) between selenium-treated groups and the control group at the identical germination stage. S1, soaked mung beans.

In the process of growth and development, plants can absorb selenium through passive diffusion and sulfate transporters [16]. After treatment with Na_2_SeO_3_ solution, the total selenium content of mung bean sprouts at different germination times increased significantly (*p* < 0.05) compared with the control (treatment with distilled water), as shown in Figure 3A. With the increase in Na_2_SeO_3_ concentration, the content of organic selenium first increased and then decreased under different germination times, which can be seen in Figure 3B. The decrease in organic selenium may be due to the toxic effect of high concentrations of Na_2_SeO_3_ on mung bean sprouts, which reduced the ability of the plants to convert inorganic selenium into organic selenium. When the exogenous selenium concentration ranged from 30 to 120 mg/L, the organic selenium content showed no significant difference (*p* > 0.05) among S1, 1 d and 2 d germination stages; however, at 3 d of germination, organic selenium content decreased significantly (*p* < 0.05). This indicated that the mung beans had completed the conversion of inorganic selenium to organic selenium in the soaking stage. The decrease in organic selenium caused by prolonged germination time may be attributed to the conversion of selenium into volatile selenides such as dimethylselenide and dimethyl diselenide [20]. The content of organic selenium in mung bean sprouts treated with Na_2_SeO_3_ reached 7.73–13.42 mg/kg, which was significantly (*p* < 0.05) increased compared with the control. This was consistent with our previous research results on germinated millet that total selenium and organic selenium could be effectively enriched after Na_2_SeO_3_ treatment [17]. Similar results were also observed in germinated barley [20], germinated soybean [6], and alfalfa sprouts [46].

#### 3.2.2. Impacts of Sodium Selenite Soaking Treatment Concentration on GABA Accumulation in Mung Bean Sprouts

Variations in GABA levels of mung bean sprouts in response to different Na_2_SeO_3_ soaking doses are visualized in Figure 3C. GABA concentrations in mung bean sprouts harvested at different germination time points showed an initial rise followed by a decline with increasing Na_2_SeO_3_ concentrations. At 90 mg/L Na_2_SeO_3_, sprouts germinated for 3 d reached the maximum GABA concentration of 1193.81 mg/kg, 3.49-fold of the control. A prior investigation [18] reported analogous observations: Na_2_SeO_3_ treatment induced prominent GABA enhancement in germinated black soybeans, and this phenomenon originated from selenium-mediated elevation in GAD activity.

#### 3.2.3. Effect of Na_2_SeO_3_ Soaking Concentration on TPC of Mung Bean Sprouts

**Figure 4:**
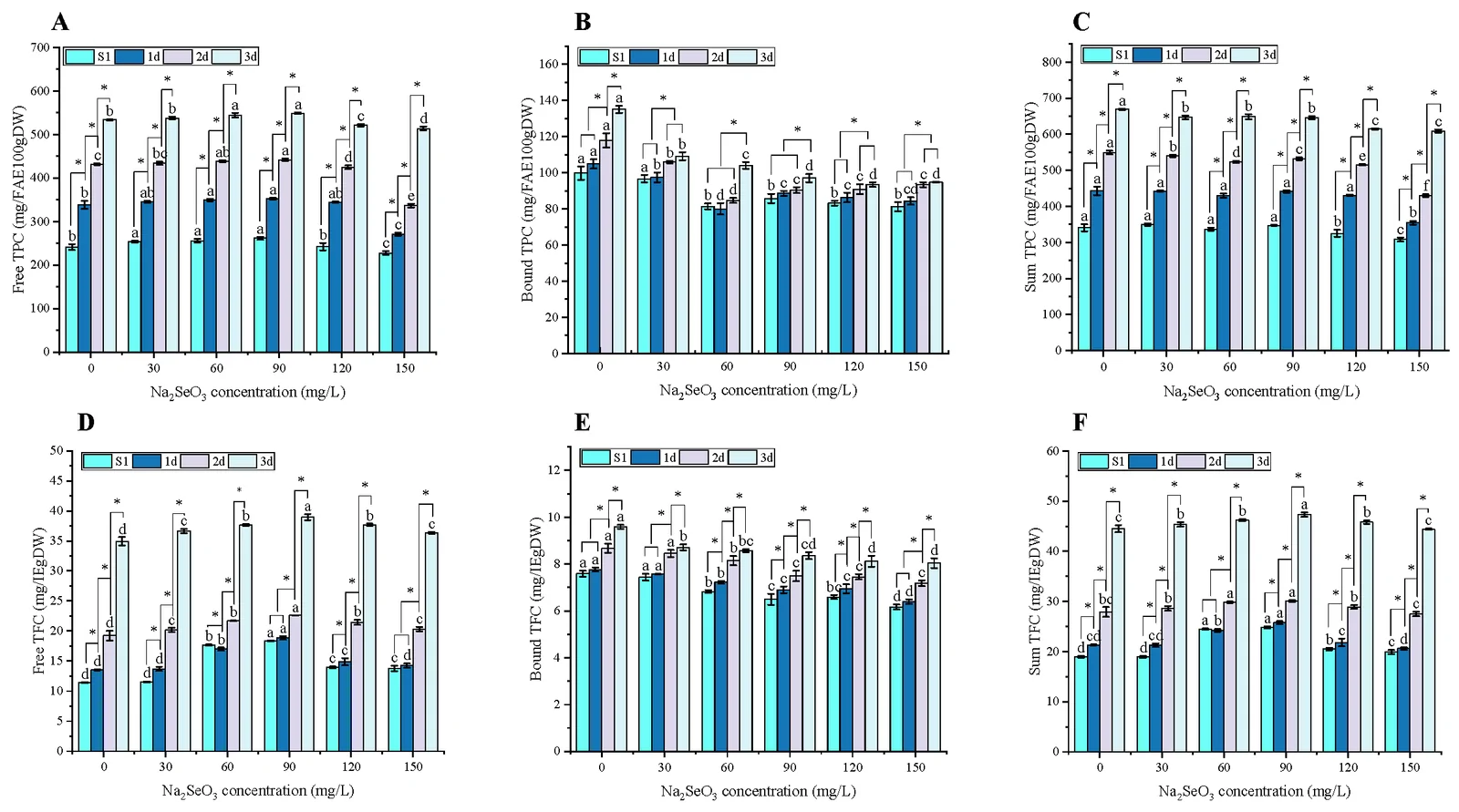
Effect of Na_2_SeO_3_ concentration on free TPC (**A**), bound TPC (**B**), sum TPC (**C**), free TFC (**D**), bound TFC (**E**), and sum TFC (**F**) of sprouted mung beans. Within the same Na_2_SeO_3_ concentration, different lowercase letters indicate significant differences (*p* < 0.05) among different germination stages. Asterisks (*) denote significant differences (*p* < 0.05) between selenium-treated groups and the control group at the identical germination stage. S1, soaked mung beans.

Elevated Na_2_SeO_3_ concentrations induced a tendency of initial accumulation followed by subsequent reduction in free TPC among mung bean sprouts harvested at different germination time points (Figure 4A). After sodium selenite exposure, bound TPC in sprouted mung beans across all germination periods dropped below the corresponding control values, as presented in Figure 4B. Variations in total TPC in response to changing Na_2_SeO_3_ concentrations are visualized in Figure 4C. When the concentration of Na_2_SeO_3_ exceeded 120 mg/L, the sum TPC of S1 and mung beans germinated for 1 d decreased significantly (*p* < 0.05). The sum TPC of mung beans germinated for 2 d and 3 d after selenium-enriched treatment decreased significantly (*p* < 0.05) compared with the control. Treatment with Na_2_SeO_3_ enhanced PAL activity, which contributed to phenolic compound accumulation and thereby increased free TPC [3]. Similar results have been reported by Islam et al. [16], that the polyphenol of wheat microgreens could be enriched after Na_2_SeO_3_ treatment. The reduction in bound TPC may be attributed to the increase in hydrolase activity after selenium treatment, resulting in the release of bound phenolic compounds, which also led to the rise in free TPC. However, total TPC did not increase under selenium treatment, because the released bound polyphenols were not fully converted to free polyphenols.

#### 3.2.4. Effect of Na_2_SeO_3_ Soaking Concentration on TFC of Sprouted Mung Beans

With rising Na_2_SeO_3_ levels, free TFC exhibited an initial rise followed by a decline across all sampled germination periods (Figure 4D). Selenium application triggered reductions in bound TFC of mung bean sprouts collected at different germination time points, as illustrated in Figure 4E. In addition, notable enhancement (*p* < 0.05) in total TFC was detected after Na_2_SeO_3_ treatment, with the maximum value attained by 3-day-old sprouts exposed to 90 mg/L Na_2_SeO_3_ solution. The increase in free TFC may arise from Na_2_SeO_3_-induced elevation in PAL, CHI and chalcone synthase (CHS) activities, which facilitated flavonoid accumulation [47]. At the same time, variations in free and bound TFC might also be linked to the accelerated liberation of bound phenolic compounds triggered by selenium treatment. Huang et al. [18] also found that Na_2_SeO_3_ treatment could lead to an increase in TFC in germinated black soybeans.

#### 3.2.5. Effect of Na_2_SeO_3_ Soaking Concentration on AKG Content of Mung Bean Sprouts

**Figure 5:**
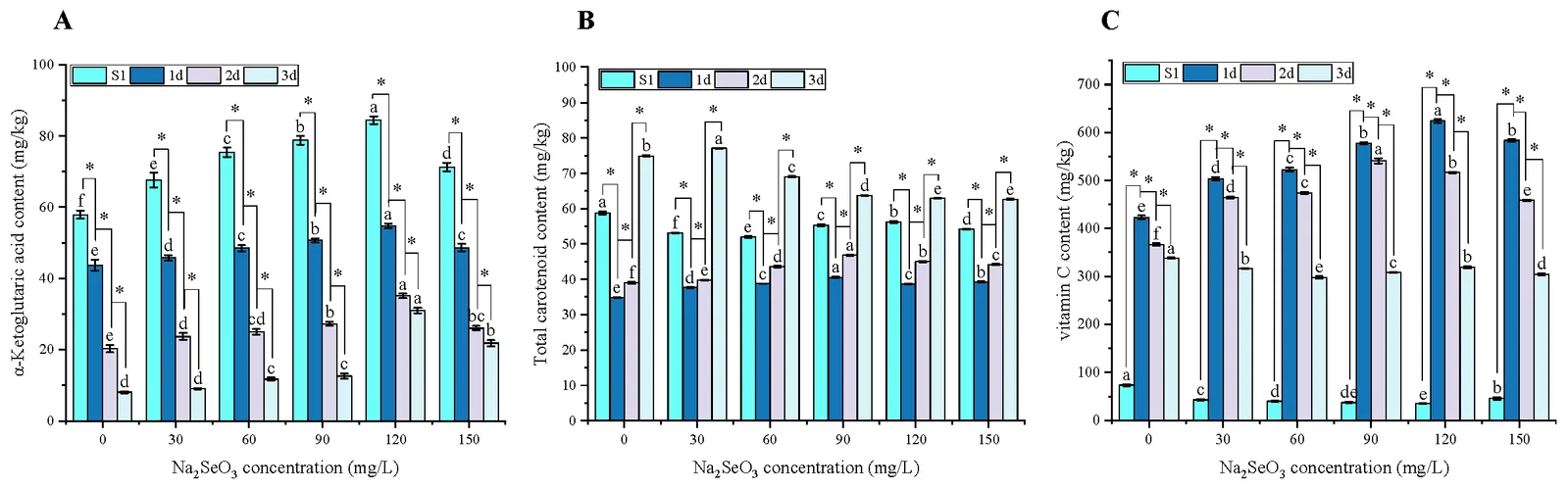
Effect of Na_2_SeO_3_ concentration on AKG content (**A**), total carotenoid content (**B**), and VC content (**C**) of sprouted mung beans. Within the same Na_2_SeO_3_ concentration, different lowercase letters indicate significant differences (*p* < 0.05) among different germination stages. Asterisks (*) denote significant differences (*p* < 0.05) between selenium-treated groups and the control group at the identical germination stage. S1, soaked mung beans.

The effect of Na_2_SeO_3_ concentration on the content of AKG is shown in Figure 5A. At different germination durations, Na_2_SeO_3_ treatment significantly (*p* < 0.05) elevated AKG concentrations, with the optimal enrichment observed at 120 mg/L. After Na_2_SeO_3_ treatment, the content of AKG in S1 and mung beans germinated for 1 d, 2 d, and 3 d increased by 45.92%, 25.26%, 73.29% and 285.46%, respectively, compared with the control. It is speculated that this may be due to selenium increasing the activity of glutamate dehydrogenase, resulting in the conversion of glutamate to AKG. Our findings indicate that Na_2_SeO_3_ treatment enhances the potential of mung bean sprouts as an AKG-rich functional food. To the best of our knowledge, this is the first report describing the effect of Na_2_SeO_3_ on AKG concentrations in germinated mung beans.

#### 3.2.6. Effect of Na_2_SeO_3_ Soaking Concentration on TCC of Mung Bean Sprouts

The effect of Na_2_SeO_3_ concentration on TCC is shown in Figure 5B. Compared with the control, Na_2_SeO_3_ treatment reduced TCC in S1, whereas appropriate Na_2_SeO_3_ concentrations increased TCC in mung bean sprouts at 1 d, 2 d and 3 d. The elevated carotenoid levels may be attributed to selenium’s ability to scavenge free radicals and suppress lipid oxidation and hydroperoxide formation in plants, thereby protecting carotenoids [48]. This agrees with previous findings [49] reporting that Na_2_SeO_3_ treatment increased carotenoid contents in broccoli sprouts. Islam et al. [16] also reported that Na_2_SeO_3_ could increase the content of carotenoids in wheat microgreens.

#### 3.2.7. Effect of Na_2_SeO_3_ Soaking Concentration on VC Content of Mung Bean Sprouts

Compared with control samples, selenium-treated S1 and 3-day mung bean sprouts showed a significant reduction (*p* < 0.05) in VC content (Figure 5C). At the same time, we also observed that selenium treatment could increase the VC content of mung bean sprouts germinated for 1 d and 2 d. The VC increase may arise because selenium in plants mitigates VC oxidative degradation induced by hydrogen peroxide and lipid peroxides [18]. Similar selenium-mediated effects on VC accumulation have been documented in soybean sprouts [50], pea sprouts [19], and radish sprouts [51,52].

### 3.3. Identification of Phenolic Compounds in Mung Bean Sprouts

**Figure 6:**
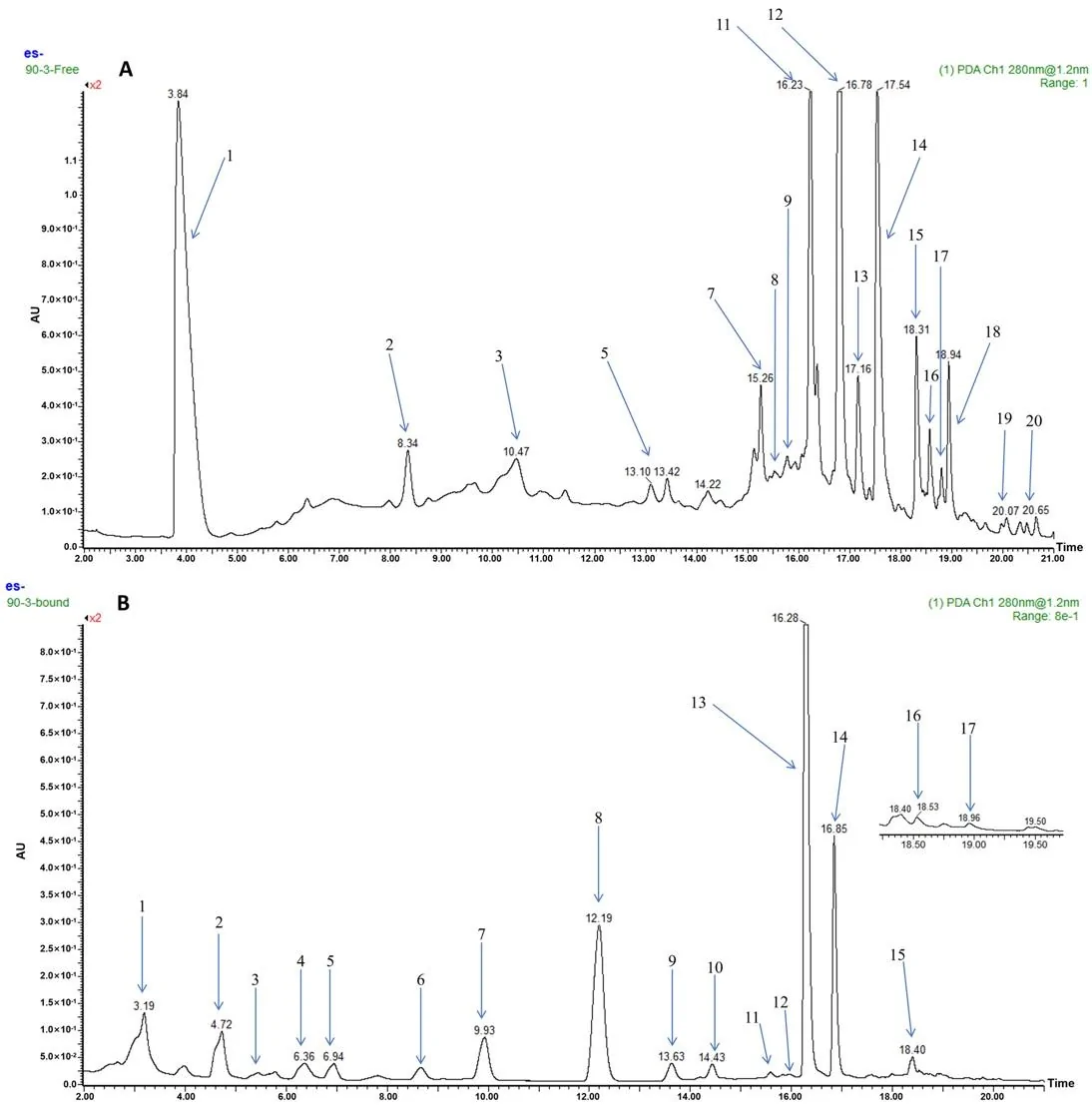
UPLC chromatograms of free (**A**) and bound (**B**) phenolics from sprouted mung beans with Na_2_SeO_3_ treatment. Detections were set at wavelength of 280 nm. Compounds were confirmed by comparing with standards or putatively identified by UV spectra, MS, and MS/MS data.

Germinating mung beans soaked in distilled water and Na_2_SeO_3_ solution exhibited largely similar phenolic fingerprints. Representative separation profiles for free (A) and bound (B) phenolic fractions from mung bean sprouts subjected to 90 mg/L Na_2_SeO_3_ exposure are illustrated in Figure 6. Identification data of free and bound phenolic analytes, including their retention times, MS and spectral information, are summarized in Tables S1 and S2, respectively.

#### 3.3.1. Free Phenolic Profiles

Based on previous findings [53], tryptophan, eriodictyol hexoside, orientin, isoorientin, vitexin, isovitexin, vitexin isomer, and isovitexin isomer were assigned to compounds **1**, **4**, **8**, **9**, **11**, **12**, **14**, **16**, and **18**, respectively. For the remaining phenolic analytes, tentative identification was performed by matching spectral features and MS^2^ fragmentation patterns against the literature data.

For compound **2**, the deprotonated ion [M − H]^−^ appeared at *m*/*z* 655. Its major MS^2^ fragmentation profile yielded signals at *m*/*z* 609 (100%), *m*/*z* 447 (60%), and *m*/*z* 285 (10%). Due to the presence of formic acid in the mobile phase, compound 2 was acidified to produce molecular ions at *m*/*z* 655. After neutral loss of one hexose and two hexoses, compound **2** produced MS/MS fragments at *m*/*z* 447 and *m*/*z* 285, respectively. Compound **2** was tentatively identified as kaempferol dihexoside by comparison with the literature [54]. Compound **3** exhibited a deprotonated molecular ion [M − H]^−^ at *m*/*z* 449 and produced a typical MS^2^ fragment at *m*/*z* 287 [M − H − 162]^−^. By comparison with the previous literature [55], compound **3** was deduced to be dihydrokaempferol hexoside. For compound **5**, the deprotonated ion [M − H]^−^ appeared at *m*/*z* 593. Its prominent MS^2^ fragmentation yielded fragment ions at *m*/*z* 503 [M − H − 90]^−^ and *m*/*z* 473 [M − H − 120]^−^. These fragmentations confirmed that compound 5 was a C-glycoside; it was tentatively identified as apigenin C-hexoside-hexoside by comparison with the prior literature [56]. Compound **6** had a molecular ion [M − H]^−^ at *m*/*z* 493 and produced a MS/MS fragment at *m*/*z* 447, which was due to the loss of one molecule of formic acid. Typical MS/MS fragments were detected at *m*/*z* 285, which was attributed to the loss of a hexoside. Compound 6 was deduced to be kaempferol 3-O-glucoside by comparison with previous research [54]. Compound **7** exhibited a deprotonated molecular ion [M − H]^−^ at *m*/*z* 593 and produced main MS^2^ fragments at *m*/*z* 431 [M − H − 162]^−^, *m*/*z* 341 [M − H − 162 − 90]^−^, and *m*/*z* 311 [M−H − 162 − 120]^−^. Compound **7** was tentatively identified as 6-C-7-O-diglucosyl-apigenin by comparison with the previous report [57]. Compound **10** showed the deprotonated ion [M − H]^−^ at *m*/*z* 477. Its characteristic fragment ions were *m*/*z* 431 [M − H − 46]^−^ and *m*/*z* 269 [M − H − 46 − 162]^−^, corresponding to sequential neutral losses of formic acid and a hexose moiety. Compound **10** was tentatively identified as apigenin-7-O-glucoside by comparison with an existing study [58]. For compound 13, the deprotonated ion [M − H]^−^ was detected at *m*/*z* 609, accompanied by a fragment signal at *m*/*z* 301 [M − H − 308]^−^. Comparison of retention time, spectral data and MS-MS fragments with the reference standard enabled its identification as rutin. Compound **15** showed a deprotonated ion [M − H]^−^ at *m*/*z* 287 and yielded characteristic MS^2^ fragments at *m*/*z* 125 [M − H − 162]^−^, and it was tentatively identified as eriodictyol [59]. Compound **17** produced a deprotonated ion [M − H]^−^ at *m*/*z* 285, with characteristic MS^2^ fragment ions at *m*/*z* 151 and *m*/*z* 133. These fragments arose from retro-Diels–Alder (RDA) cleavage, a characteristic fragmentation pathway of flavonoids. Compound 17 was tentatively identified as luteolin [60]. Compound **19** produced a molecular ion [M − H]^−^ at *m*/*z* 301, MS^2^ fragment ion at *m*/*z* 151 [M − H − C_8_H_6_O_3_]^−^, which was inferred to be quercetin [61]. Compound **20** displayed the deprotonated ion [M − H]^−^ at *m*/*z* 269 and a dominant MS^2^ fragment at *m*/*z* 133 [M − H − 136]^−^. It was identified as genistein by comparison with a previous study [62].

#### 3.3.2. Bound Phenolic Profiles

According to the previous literature [53], compounds **1**, **2**, **3**, **4**, **5**, **6**, **7**, **8**, **11**, **12**, **13**, **14**, **16**, and **17** were identified as hydroxyphenyllactic acid, protocatechuic aldehyde, *p*-hydroxybenzoic acid, methyl gallate, *p*-hydroxybenzaldehyde, 2,4-dihydroxybenzoic acid, phenylacetic acid, *trans*-*p*-coumaric acid, orientin, isoorientin, vitexin, isovitexin, vitexin isomer, and isovitexin isomer, respectively.

Both compound **8** and compound **9** showed the deprotonated ion [M − H]^−^ at *m*/*z* 163 and yielded the dominant fragment ion at *m*/*z* 119 (100% relative abundance). Based on previously reported results [24], compound **9** was putatively annotated as *cis*-*p*-coumaric acid. The identity of compound **10** was confirmed as ferulic acid by matching its retention behavior, spectral profiles, and MS^2^ fragments with an authentic external standard. Within the free-phenolic pool, compound **15** was hypothesized to correspond to eriodictyol.

### 3.4. Effect of Selenium Biofortification on the Content of Individual Phenolic Compounds of Mung Bean Sprouts

#### 3.4.1. Individual Free Phenolic Compounds

Variations in the individual free phenolic profiles of mung beans over different germination periods are listed in Table S3. Vitexin and isovitexin remained the primary phenolic compounds in sprouting mung bean samples. Extended germination duration induced the depletion of free vitexin and isovitexin. Similar findings were reported by Tang et al. [63], who found that prolonged germination reduced vitexin and isovitexin concentrations. This trend is presumably associated with the metabolic transformation of vitexin and isovitexin into other derivatives during germination. Prolonging germination significantly increased vitexin-*O*-rhamnoside, which reached its maximum on the third day of germination. Kaempferol dihexoside, apigenin-*C*-hexoside-hexoside, eriodictyol, quercetin, and genistein were detected on the first day of germination and gradually accumulated with the extension of the germination process. As reported in a prior investigation [64], a remarkable rise in quercetin levels was observed in highland barley upon germination. Rutin was detected on the second day of mung bean germination, and the extension of the germination period could effectively increase the level of rutin. Consistent with the present findings, Zhang et al. [65] noted that germination brought about higher rutin levels within buckwheat seeds. Orientin, isoorientin, and vitexin isomer in mung beans continuously decreased with increasing germination time, whereas isovitexin isomer exhibited the opposite trend. The accumulation of phenolic compounds may be attributed to elevated PAL activity during germination [31].

The contents of vitexin, isovitexin, vitexin-*O*-rhamnoside, rutin, eriodictyol, quercetin, genistein, isoorientin, apigenin-*C*-hexoside-hexoside, and kaempferol dihexoside at different germination stages were significantly (*p* < 0.05) increased by Na_2_SeO_3_ treatment. Comparable findings were reported in the literature [7], where selenium increased vitexin and isovitexin levels in mung bean sprouts of certain genotypes. Pannico et al. [66] also found that selenium could increase the level of rutin in microgreens. Following Na_2_SeO_3_ treatment, dihydrokaempferol hexoside was significantly increased (*p* < 0.05) in mung beans germinated for 1 d, 2 d, and 3 d. The accumulation of phenolic compounds may arise from selenium-induced increases in PAL activity [67]. Apigenin-7-O-glucoside was detected only in mung beans germinated for 1 d after Na_2_SeO_3_ treatment, reaching 34.85 mg/kg. Selenium treatment reduced orientin and vitexin isomer contents in mung beans germinated for 1 d, 2 d, and 3 d.

#### 3.4.2. Individual Bound Phenolic Compounds

Variations in individual bound phenolic compound contents among mung bean samples from distinct germination periods are presented in Table S4. Vitexin and isovitexin still constituted the predominant phenolic substances within the bound fraction. Accumulation of bound vitexin and isovitexin was observed during germination, which may originate from interconversion of their free forms. The contents of *p*-hydroxybenzoic acid, methyl gallate, *p*-hydroxybenzaldehyde, *trans*-*p*-coumaric acid, *cis*-*p*-coumaric acid, ferulic acid, orientin, and isoorientin increased continuously during the selected germination period. Similar trends have been reported in germinated millet, where germination induced accumulation of bound *trans*-*p*-coumaric acid, *cis*-*p*-coumaric acid, and ferulic acid [23]. Ma et al. [68] reported that germination increased bound *p*-hydroxybenzoic acid content in soybeans. Hydroxyphenyllactic acid and protocatechuic aldehyde continuously increased in the early germination stage but declined at day 3 of germination. The accumulation of these bound phenolic compounds may result from elevated PAL activity during germination.

Na_2_SeO_3_ treatment reduced the contents of bound vitexin and isovitexin at different germination stages. Following Na_2_SeO_3_ treatment, vitexin contents decreased by 33.54%, 8%, 0.4%, and 13.29% in S1 and mung beans germinated for 1 d, 2 d, and 3 d, respectively. At the same time, isovitexin decreased by 11.23%, 13.85%, 1.06%, and 11.21%, respectively. *Trans*-*p*-coumaric acid was the predominant phenolic acid in germinated mung beans, and its content was significantly reduced by selenium treatment. Na_2_SeO_3_ also led to a decrease in ferulic acid content. This reduction in bound phenolic compounds may be attributed to Na_2_SeO_3_-induced elevation of hydrolase activity during germination, which promoted the release of bound phenolic compounds. Methyl gallate, 2,4-dihydroxybenzoic acid, and *cis*-*p*-coumaric acid were significantly higher on day 3 of germination under selenium treatment relative to the control. Soaking in Na_2_SeO_3_ solution significantly (*p* < 0.05) increased eriodictyol content in sprouted mung beans.

## 4. Discussion

From a nutritional perspective, selenium-biofortified mung bean sprouts obtained via Na_2_SeO_3_ presoaking exhibited considerable capacity to accumulate organic selenium, which is recognized to possess higher human bioavailability compared with inorganic selenium forms [69]. In this study, appropriate sodium selenite treatments yielded organic selenium contents ranging from 7.73 to 13.42 mg/kg DW in mung bean sprouts. According to FAO/WHO recommendations, the daily dietary selenium requirement for adults is 50–60 μg per day [2]. Although direct consumption of selenium-enriched mung bean sprouts cannot fully meet daily selenium demands alone, such edible sprouts can serve as valuable dietary contributors to narrow selenium nutritional gaps for selenium-deficient populations when incorporated into regular plant-based diets [66]. It should be noted that excessive selenium intake carries potential toxic risks; hence, controlling the exogenous selenite concentration during sprouting production is essential to balance selenium enrichment and food safety, which is consistent with previous reports on selenium-fortified pulse sprouts. The conversion of inorganic selenium absorbed by mung bean seeds into organic selenium fractions further supports the feasibility of producing selenium-enriched vegetable food ingredients through simple germination-based biofortification.

Beyond selenium enrichment, the simultaneous elevation of multiple health-related metabolites further improves the overall nutritional quality of edible mung bean sprouts after suitable selenium biofortification. Selenium treatment not only increased GABA and AKG contents, with documented benefits for human nervous system and energy metabolism, but also remodeled phenolic profiles by shifting polyphenols from bound fractions toward free fractions. Free phenolics and flavonoids generally show superior bioaccessibility during gastrointestinal digestion relative to bound phenolic forms that tightly bind to cell-wall components [5]. As the predominant flavonoid constituents in mung bean sprouts, vitexin and isovitexin were significantly enhanced in their free forms under selenium treatment. Collectively, selenium-biofortified 3-day-harvested mung bean sprouts combine dietary selenium supply with abundant free-form phenolic compounds, GABA, AKG, VC and carotenoids. This multi-component nutritional advantage highlights its application prospect as an innovative functional vegetable ingredient rather than merely representing physiological metabolic variation during seed germination.

## 5. Conclusions

This study demonstrated that germination elevated free- and bound-form total phenolic (TPC) and total flavonoid contents (TFC) as well as total carotenoids (TCC), GABA, AKG, and VC in mung bean sprouts. Vitexin and isovitexin, the predominant flavonoids in both free and bound phenolic fractions, decreased in their free forms but accumulated in bound forms during germination. *Trans*-*p*-coumaric acid, the major phenolic acid in germinating mung beans, continuously built up over prolonged sprouting. Mung bean sprouts presoaked in Na_2_SeO_3_ efficiently accumulated selenium, revealing their practical potential as edible plant-based sources for dietary selenium supplementation. Appropriate Na_2_SeO_3_ treatment further enhanced GABA, free-TPC, free-TFC, AKG, TCC, and VC levels and shifted phenolic partitioning toward free fractions by increasing free vitexin and isovitexin while lowering their bound counterparts. Collectively, these outcomes offer theoretical foundations for producing selenium-enriched mung bean sprout foods with desirable functional nutritional attributes. Since the present work focused primarily on selenium accumulation and changes in functional metabolites induced by Na_2_SeO_3_ treatment, further investigations into the bioavailability of selenium and these bioactive constituents are warranted. Such follow-up work will deliver more comprehensive evidence to support the industrial development of functional selenium-fortified mung-bean-sprout food ingredients.

## Data Availability

The original contributions presented in this study are included in the article/Supplementary Material. Further inquiries can be directed to the corresponding author.

## Supplementary Materials

The following supporting information can be downloaded at: https://www.mdpi.com/article/10.3390/foods15203695/s1, Table S1: Individual phenolic compounds identified using UPLC-ESI-MS/MS in the methanol extracts of sprouted mung beans; Table S2: Individual phenolic compounds identified using UPLC-ESI-MS/MS in the bound fraction of sprouted mung beans; Table S3: Content of individual phenolic compounds (mg/kg) in free phenolic extracts of sprouted mung beans; Table S4: Content of individual phenolic compounds (mg/kg) in bound phenolic extracts of sprouted mung beans.
