# Supplementary material for: Selenium Biofortification Enhances Functional Bioactive Components and Nutritional Quality of Edible Mung Bean Sprouts During Germination
Source: Foods. 2026 Oct 17;15(20):3695. doi: 10.3390/foods15203695 (PMC13704497; doi:10.3390/foods15203695)
Supplement: Supplementary file 1 [file foods-15-03695-s001.zip › foods-4569918-Supplementary.pdf]

Electronic Supplementary Information

Selenium Biofortification Enhances Functional Bioactive Components and Nutritional Quality of Edible Mung Bean Sprouts During Germination

Xiangxiang He [1,2], Shibin Yu [1,2], Bo Zhang [3,4], Runqiang Yang [5], YiZhuo Han [1], Hongwei Hao [1], and Jinle Xiang [1,2,*]

[1] College of Food and Bioengineering, Henan University of Science and Technology, Luoyang 471023, China; xianghe2009@126.com (X.H.); 18837649619@163.com (S.Y.); han0701042026@163.com (Y.H.); h138025@163.com (H.H.)

[2] Henan International Joint Laboratory of Food Green Processing and Safety Control, Henan University of Science and Technology, Luoyang 471023, China

[3] School of Basic Medical Sciences, Zunyi Medical University, Zunyi 563000, China; bozhang.zum@163.com

[4] Zunyi Center for Disease Control and Prevention (Zunyi Health Supervision Bureau), Zunyi 563000, China

[5] College of Food Science & Technology, Nanjing Agricultural University, Nanjing 210095, China; yangrq@njau.edu.cn

[*]Correspondence: xjl5013@haust.edu.cn; Tel.: +86-152-3791-6981

Table S1. Individual phenolic compounds identified using UPLC-ESI-MS/MS in the methanol extracts of sprouted mung bean.

No.	Retention time	[M-H]− / (m/z)	UV λ max (nm)	Formula	m/z of main fragments (relative intensity, %), MS/MS	Compound identified	0d	1d	2d	3d
**1**	3.84	203	279	$C_{11}H_{12}N_2O_2$	142(70),116(100),74(60)	Tryptophan	+	+	+	+
**2**	8.34	655	257	$C_{27}H_{30}O_{16}$	609(100),447(60),309(10),285(10)	Kaempferol dihexoside	-	+	+	+
**3**	10.47	449	292	$C_{21}H_{22}O_{11}$	449(90),287(80),179(40)	Dihydrokaempferol hexoside	+	+	+	+
**4**	12.91	449	292	$C_{21}H_{22}O_{11}$	287(10),197(50),167(100),125(10)	Eriodictyol hexoside	+	+	+	-
**5**	13.10	593	272/325	$C_{27}H_{30}O_{15}$	593(100),503(10),473(40),341(10),311(10)	Apigenin-C-hexoside-hexoside	-	+	+	+
**6**	13.67	493	258	$C_{21}H_{20}O_{10}$	447(40),327(40),309(40),285(100)	kaempferol 3-O-glucoside	-	+	-	-
**7**	15.28	593	271/325	$C_{27}H_{30}O_{15}$	593(70),431(10),341(30),311(100),269(10)	6-C-7-O-diglucosyl-apigenin	+	+	+	+
**8**	15.65	447	270/349	$C_{21}H_{20}O_{11}$	357(30),327(100)	Orientin	+	+	+	+
**9**	15.82	447	268/328	$C_{21}H_{20}O_{11}$	357(100),327(95)	Isoorientin	+	+	+	+
**10**	16.08	477	260	$C_{21}H_{20}O_{10}$	431(100),269(70)	Apigenin-7-O-glucoside	-	+	-	-
**11**	16.36	431	268/338	$C_{21}H_{20}O_{10}$	341(30),311(100),283(10)	Vitexin[a]	+	+	+	+
**12**	16.94	431	269/338	$C_{21}H_{20}O_{10}$	341(50),311(100),283(10)	Isovitexin[a]	+	+	+	+
**13**	17.16	609	256/353	$C_{27}H_{30}O_{16}$	301(10)	Rutin[a]	-	-	+	+
**14**	17.73	577	270/335	$C_{27}H_{30}O_{14}$	341(20),311(80)	Vitexin-O-rhamnoside	+	+	+	+
**15**	18.31	287	287	$C_{15}H_{12}O_5$	177(10),161(20),125(100),	Eriodictyol	-	+	+	+
**16**	18.56	431	270/335	$C_{21}H_{20}O_{10}$	341(50),311(100),283(10)	Vitexin isomer	+	+	+	+
**17**	18.70	285	258	$C_{15}H_{10}O_6$	217(100),175(50),151(15),133(15)	Luteolin	-	+	+	-
**18**	18.94	431	270/319	$C_{21}H_{20}O_{10}$	341(90),311(100),283(10)	Isovitexin isomer	+	+	+	+
**19**	20.02	301	287	$C_{15}H_{10}O_7$	285(10),177(10),165(100),151(10)	Quercetin	+	+	+	+
**20**	20.10	269	260	$C_{15}H_{10}O_5$	225(10),133(10)	Genistein	-	+	+	+

[a] Identification of the compounds was confirmed using an authentic standard. All other compounds were tentatively identified by comparing their mass spectral characteristics with those reported in the literature. "+" indicates that it was detected and "-" indicates that it is not detected.

Table S2. Individual phenolic compounds identified using UPLC-ESI-MS/MS in the bound fraction of sprouted mung bean.

No.	Retention time	[M-H]− / (m/z)	UV λ max (nm)	Formula	m/z of main fragments (relative intensity, %), MS/MS	Compound identified	0d	1d	2d	3d
**1**	3.19	181	291	C9H10O4	153(70),135(100)	Hydroxyphenyllactic acid	+	+	+	+
**2**	4.72	137	280	C7H5O3	119(60),108(80),93(10)	Protocatechuic aldehyde[a]	+	+	+	+
**3**	5.48	137	255	C7H5O3	93(100)	*p*-hydroxybenzoic acid[a]	+	+	+	+
**4**	6.36	183	281	C8H8O5	165(40),137(100),125(80)	Methyl gallate	+	+	+	+
**5**	6.94	121	283	$C_7H_5O_2$	-	*p*-hydroxybenzaldehyde[a]	+	+	+	+
**6**	8.69	153	291	C7H6O4	125(90),109(40),107(100)	2,4-dihydroxybenzolic acid[a]	+	+	+	+
**7**	9.93	135	276	C8H8O2	120(50),117(10),92(100)	Phenylacetic acid	+	+	+	+
**8**	12.19	163	309	C9H7O3	119(100)	*trans*-*p*-coumaric acid[a]	+	+	+	+
**9**	13.63	163	293	C9H7O3	119(100)	*cis*-*p*-coumaric acid	-	+	+	+
**10**	14.43	193	280/323	C10H9O4	178(70),134(100)	Ferulic acid[a]	-	+	+	+
**11**	15.59	447	270/349	$C_{21}H_{20}O_{11}$	357(30),327(100)	Orientin	+	+	+	+
**12**	15.82	447	268/328	$C_{21}H_{20}O_{11}$	357(100),327(90)	Isoorientin	+	+	+	+
**13**	16.28	431	268/338	C21H20O10	341(30),311(100),283(10)	Vitexin[a]	+	+	+	+
**14**	16.85	431	269/328	C21H20O10	341(50),311(100),283(10)	Isovitexin[a]	+	+	+	+
**15**	18.40	287	286	C15H12O6	177(20),161(30),125(100)	Eriodictyol	-	+	+	+
**16**	18.53	431	270/335	$C_{21}H_{20}O_{10}$	341(50),311(100),283(10)	Vitexin isomer	+	+	+	+
**17**	18.96	431	270/319	$C_{21}H_{20}O_{10}$	341(90),311(100),283(10)	Isovitexin isomer	+	+	+	+

[a] Identification of the compounds was confirmed using an authentic standard. All other compounds were tentatively identified by comparing their mass spectral characteristics with those reported in the literature. “+” indicates that it was detected and “-” indicates that it is not detected.

Table S3. Content of individual phenolic compounds (mg/kg) in free phenolic extracts of sprouted mung bean.

No	Phenolic compounds	Treatment	0d	1d	2d	3d
**1**	Kaempferol dihexoside	Control	n.d.	3.87±0.12Bc	8.75±0.19Bb	22.41±0.24Ba
		90mg/L Na_2SeO_3	n.d.	19.27±0.30Ac	22.09±0.23Ab	46.55±0.86Aa
**2**	Dihydrokaempferol hexoside	Control	10.26±0.22Ad	14.59±0.42Bc	20.95±0.37Bb	22.88±0.24Ba
		90mg/L Na_2SeO_3	5.92±0.17Bd	22.38±0.40Ac	26.27±1.40Ab	29.87±1.16Aa
**3**	Eriodictyol hexoside	Control	100.28±1.03Aa	16.77±0.43Bb	9.22±0.58Bc	n.d.
		90mg/L Na_2SeO_3	102.01±0.41Aa	43.18±0.93Ab	25.12±0.64Ac	n.d.
**4**	Apigenin-C-hexoside-hexoside	Control	n.d.	1.38±0.06Bc	8.66±0.27Bb	34.88±0.41Ba
		90mg/L Na_2SeO_3	n.d.	6.02±0.17Ac	15.12±0.33Ab	38.53±0.39Aa
**5**	Kaempferol 3-O-glucoside	Control	n.d.	3.12±0.03A	n.d.	n.d.
		90mg/L Na_2SeO_3	n.d.	3.10±0.04A	n.d.	n.d.
**6**	6-C-7-O-diglucosyl-apigenin	Control	1.43±0.05Bd	29.10±0.59Aa	17.96±0.22Bb	15.84±0.28Bc
		90mg/L Na_2SeO_3	1.62±0.06Ad	20.79±0.44Bc	25.84±0.47Ab	46.41±1.22Aa
**7**	Orientin	Control	16.60±0.35Ba	16.07±0.43Aa	14.33±0.27Ab	9.32±0.41Ac
		90mg/L Na_2SeO_3	19.27±0.38Aa	13.99±0.28Bb	10.30±0.31Bc	9.05±0.14Ad
**8**	Isoorientin	Control	29.43±0.40Ba	23.47±0.30Bb	16.32±0.18Bc	14.47±0.27Bd
		90mg/L Na_2SeO_3	31.39±0.29Aa	24.67±0.24Ab	19.03±0.25Ac	15.85±0.24Ad
**9**	Apigenin-7-O-glucoside	Control	n.d.	n.d.	n.d.	n.d.
		90mg/L Na_2SeO_3	n.d.	34.85±0.40	n.d.	n.d.
**10**	Vitexin	Control	664.85±4.28Ba	333.39±2.31Bb	332.76±0.92Bb	332.93±2.48Bb
		90mg/L Na_2SeO_3	716.31±8.34Aa	515.69±4.40Ab	395.89±2.12Ac	377.02±2.98Ad
**11**	Isovitexin	Control	328.88±1.53Ba	202.42±1.06Bb	202.62±1.04Bb	202.12±1.05Bb
		90mg/L Na_2SeO_3	346.40±1.47Aa	335.65±0.97Ab	244.58±1.22Ac	219.95±1.57Ad
**12**	Rutin	Control	n.d.	n.d.	14.59±0.41Bb	108.91±2.86Ba
		90mg/L Na_2SeO_3	n.d.	n.d.	15.30±0.16Ab	142.75±2.75Aa

**13**	Vitexin-O-rhamnoside	Control	28.85±0.40Bd	244.65±2.60Bc	321.45±1.52Bb	377.30±2.27Ba
		90mg/L Na_2SeO_3	32.89±0.51Ad	330.56±2.68Ac	335.43±0.40Ab	413.64±2.94Aa
**14**	Eriodictyol	Control	n.d.	8.77±0.23Bc	18.82±0.43Bb	50.66±1.60Ba
		90mg/L Na_2SeO_3	n.d.	66.45±1.60Ac	70.37±0.51Ab	74.35±1.73Aa
**15**	Vitexin isomer	Control	7.70±0.12Ba	6.87±0.34Ab	6.56±0.13Ab	5.32±0.32Ac
		90mg/L Na_2SeO_3	8.33±0.16Aa	5.68±0.32Bb	4.27±0.17Bc	4.09±0.10Bc
**16**	Luteolin	Control	n.d.	4.82±0.04Bb	5.61±0.12a	n.d.
		90mg/L Na_2SeO_3	n.d.	19.33±0.44A	n.d.	n.d.
**17**	Isovitexin isomer	Control	8.83±0.19Ac	8.91±0.25Ac	12.05±0.11Bb	15.38±0.17Ba
		90mg/L Na_2SeO_3	9.14±0.38Ac	9.29±0.23Ac	12.61±0.12Ab	18.61±0.14Aa
**18**	Quercetin	Control	n.d.	2.94±0.06Bc	4.35±0.16Bb	5.37±0.09Ba
		90mg/L Na_2SeO_3	n.d.	4.71±0.30Ac	6.65±0.17Ab	8.08±0.29Aa
**19**	Genistein	Control	n.d.	0.95±0.04Bc	1.15±0.04Bb	1.66±0.10Ba
		90mg/L Na_2SeO_3	n.d.	1.61±0.11Ac	3.04±0.22Ab	7.56±0.20Aa

Different lowercase and uppercase letters are used to represent the significant differences ($p<0.05$) between rows and columns, respectively. n.d., not detected. Results are expressed as mean ± SD.

Table S4. Content of individual phenolic compounds (mg/kg) in bound phenolic extracts of sprouted mung bean.

No	Phenolic compounds	Treatment	0d	1d	2d	3d
**1**	Hydroxyphenyllactic acid	Control	21.81±0.64Ac	24.46±0.55Bb	26.91±0.51Aa	26.86±0.42Aa
		90mg/L Na_2SeO_3	20.07±0.55Bd	26.05±0.62Ab	28.11±0.81Aa	23.18±0.33Bc
**2**	Protocatechuic aldehyde	Control	11.05±0.29Ac	11.85±0.53Ab	12.87±0.36Aa	12.51±0.27Aab
		90mg/L Na_2SeO_3	10.63±0.34Ac	11.57±0.05Ab	12.29±0.27Aa	12.02±0.25Aab
**3**	*p*-hydroxybenzoic acid	Control	1.23±0.05Ad	1.77±0.02Ac	2.03±0.04Ab	3.79±0.04Aa
		90mg/L Na_2SeO_3	1.00±0.04Bd	1.74±0.02Ac	2.10±0.05Ab	2.32±0.04Ba
**4**	Methyl gallate	Control	1.83±0.04Ad	2.42±0.02Ac	3.30±0.05Ab	3.96±0.04Ba
		90mg/L Na_2SeO_3	1.63±0.03Bd	2.05±0.06Bc	2.39±0.03Bb	6.78±0.10Aa
**5**	*p*-hydroxybenzaldehyde	Control	2.44±0.05Ad	2.80±0.06Bc	3.21±0.08Bb	4.32±0.06Ba
		90mg/L Na_2SeO_3	2.48±0.05Ad	3.49±0.05Ac	4.16±0.08Ab	4.98±0.10Aa
**6**	2,4-dihydroxybenzolic acid	Control	3.51±0.02Ab	3.59±0.07Ab	4.07±0.08Aa	3.08±0.07Bc
		90mg/L Na_2SeO_3	3.35±0.02Bc	3.32±0.03Bc	3.53±0.06Bb	4.41±0.08Aa
**7**	Phenylacetic acid	Control	12.25±0.04Bd	13.79±0.04Bc	15.72±0.11Ab	17.44±0.10Aa
		90mg/L Na_2SeO_3	12.81±0.06Ac	15.60±0.03Aa	15.70±0.11Aa	14.59±0.09Bb
**8**	*trans*-*p*-coumaric acid	Control	8.97±0.11Ad	20.51±0.16Ac	36.70±0.40Ab	61.76±0.62Aa
		90mg/L Na_2SeO_3	4.43±0.06Bd	8.04±0.12Bc	26.30±0.55Bb	57.40±0.79Ba
**9**	*cis*-*p*-coumaric acid	Control	n.d.	1.74±0.05Ac	2.54±0.14Ab	3.14±0.06Ba
		90mg/L Na_2SeO_3	n.d.	1.27±0.04Bc	1.77±0.05Bb	3.61±0.06Aa
**10**	Ferulic acid	Control	n.d.	5.46±0.04Ac	10.98±0.27Ab	23.87±0.36Aa
		90mg/L Na_2SeO_3	n.d.	5.38±0.19Ac	9.68±0.22Bb	22.39±0.38Ba
**11**	Orientin	Control	2.48±0.05Ad	2.62±0.03Ac	2.79±0.08Ab	2.98±0.09Aa
		90mg/L Na_2SeO_3	0.75±0.05Bc	1.48±0.07Bb	1.59±0.09Bb	2.89±0.07Aa
**12**	Isoorientin	Control	0.38±0.03Ac	0.85±0.01Ab	0.96±0.09Aa	1.04±0.02Aa
		90mg/L Na_2SeO_3	0.41±0.01Ad	0.53±0.01Bc	0.69±0.03Bb	1.03±0.02Aa

**13**	Vitexin	Control	387.27±2.27Ad	500.25±0.94Ac	555.34±3.21Ab	570.60±3.84Aa
		90mg/L Na_2SeO_3	257.39±3.11Bd	460.18±3.71Bc	552.94±4.01Aa	494.74±3.80Bb
**14**	Isovitexin	Control	30.19±0.46Ac	42.23±0.50Ab	47.07±0.95Aa	45.94±0.69Aa
		90mg/L Na_2SeO_3	26.80±0.72Bd	36.18±0.68Bc	46.57±0.70Aa	40.79±0.66Bb
**15**	Eriodictyol	Control	n.d.	5.96±0.24Bb	5.36±0.14Bc	7.39±0.09Ba
		90mg/L Na_2SeO_3	n.d.	11.62±0.36Aa	7.41±0.23Ac	9.29±0.20Ab
**16**	Vitexin isomer	Control	1.19±0.07Ad	1.30±0.04Ac	1.43±0.04Ab	1.97±0.06Aa
		90mg/L Na_2SeO_3	0.64±0.05Bc	0.82±0.03Bb	0.92±0.05Bb	2.10±0.08Ba
**17**	Isovitexin isomer	Control	1.87±0.09Aa	1.58±0.08Ab	1.06±0.04Ac	1.53±0.05Ab
		90mg/L Na_2SeO_3	0.92±0.06Bb	0.81±0.04Bc	0.97±0.06Ab	1.25±0.05Ba

Different lowercase and uppercase letters are used to represent the significant differences ($p<0.05$) between rows and columns, respectively. n.d., not detected. Results are expressed as mean ± SD.
